# Prevalence of Morton’s toe:  a systematic review and meta-analysis

**DOI:** 10.12688/f1000research.180137.1

**Published:** 2026-05-04

**Authors:** Zainab Mohsen Hasan Habib, Abdulaziz Nawaf Al-Qaed, Anas Abdulmenen Yahya, Mohamed Abdulla Al-Balushi, Amer Almarabheh, Wael Amin Nasr El-Din, Bhagath Kumar Potu

**Affiliations:** 1 College of Medicine and Health Sciences, Arabian Gulf University, Manama, Bahrain; 2 College of Medicine and Health Sciences, Arabian Gulf University, Manama, Bahrain; 3 College of Medicine and Health Sciences, Arabian Gulf University, Manama, Bahrain; 4 College of Medicine and Health Sciences, Arabian Gulf University, Manama, Bahrain; 5 Department of Family and Community Medicine, College of Medicine and Health Sciences, Arabian Gulf University, Manama, Bahrain; 6 Department of Anatomy, College of Medicine and Health Sciences, Arabian Gulf University, Manama, Bahrain; 7 Department of Anatomy, College of Medicine and Health Sciences, Arabian Gulf University, Manama, Bahrain

**Keywords:** Morton’s toe; prevalence; associated risks; populations.

## Abstract

**Background:**

When the second toe is longer than the big toe, it is referred to as Morton’s toe. Although the occurrence of Morton’s toe is considered normal and seen across many populations, its prevalence and associated risks have not been sufficiently explored. Some studies reported a very high occurrence of Morton’s toe, whereas others reported it to be low. These differences in prevalence of Morton’s toe and lack of a standard dataset regarding the associated risks of Morton’s toe made us perform a systematic review and meta-analysis on the prevalence of Morton’s toe.

**Methods:**

A systematic literature search using PRISMA guidelines was performed in databases such as Google Scholar, MEDLINE, PubMed, SciELO, and ScienceDirect using search terms such as “Morton’s Toe” AND “Risks” AND “Prevalence,” which yielded 935 article links.

**Results:**

Of the five studies that met the inclusion criteria comprising 2636 feet were in the range of 16–90 years age. Our pooled analysis revealed that the prevalence of Morton’s toe was 40% and in different populations, it was ranging from 28–66%. Statistical heterogeneity among studies was extremely high, with a heterogeneity of I ^2^ = 97.5% (p < 0.0001), indicating that most of the variability in prevalence estimates was due to real differences between studies rather than sampling error alone.

**Conclusion:**

This study provides a comprehensive picture of the prevalence of Morton’s toe, which varies from population-to-population and is associated with certain risks such as overpronation (turning inward) of the foot, bunion deformity, hammertoe, and plantar fasciitis.

**Open Science Framework (OSF) registration:** https://doi.org/10.17605/OSF.IO/BGKY2.

## Background

**Figure 1.**
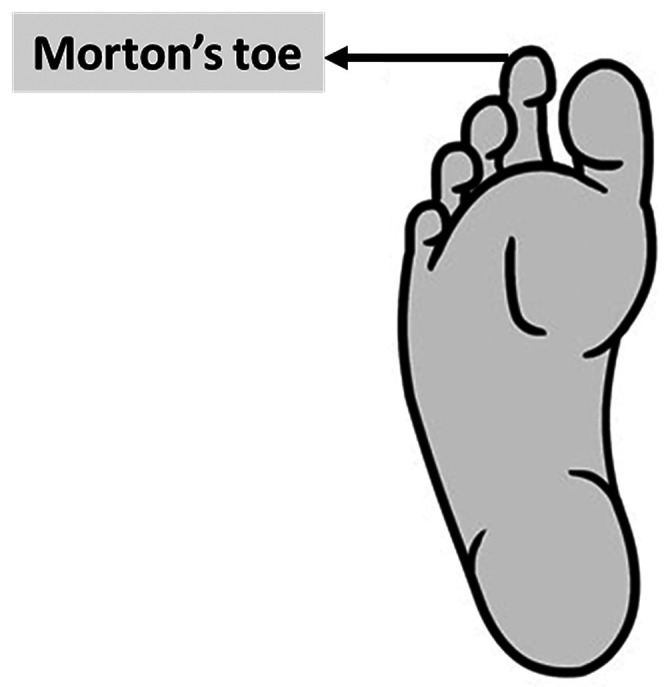
Showing the long 2 ^nd^ toe (Morton’s toe).

The foot consists of five toes, each having three phalanges, proximal, intermediate and distal, except the big toe, which is made up of only the proximal and distal phalanges. ^1^ Despite this, the big toe is usually longer than the length of second toe due to the fact that the first metatarsal bone is longer than the second metatarsal bone. This normal feature of the foot is very important for maintaining biomechanical support and balance while walking. In contrast, in some cases, the big toe could be shorter than the second toe, and when the second toe is longer than the big toe, it is called Morton’s toe ( Figure 1). This structural anomaly was first observed by British anthropologist James Park Harrison in 1864 and was later described by the American orthopedic surgeon, Dudley Morton. ^2^

Some studies even argue that Morton’s toe as Greek toe has been linked to genetics and certain races. The occurrence of Morton’s toe varies considerably across different populations. Some studies reported a high occurrence of Morton’s toe, while others reported a none-to-low occurrence. These differences in prevalence may be attributed to race, ethnicity, and the different assessment methods used to quantify the frequency of occurrence. The lack of consensus and a standard dataset regarding the true distribution of Morton’s toe makes it an excellent research field of topic to explore. Moreover, studies investigating Morton’s toe have reported many associated risks that can occur as a result of this structural variation. This is primarily due to the alteration of the foot biomechanics, which results in an excess load being transferred onto the second toe instead of the big toe, leading to an imbalance in pressure distribution and unstable gait, which leads to the formation of calluses, hammertoe, metatarsalgia, bunion deformity, pronation of the foot, and plantar fasciitis. As a result of the variability in reported prevalence and associated risks in different studies, an extensive review of available reports must be conducted and analyzed to gain a better understanding of Morton’s toe.

## Methods

### Search strategy & inclusion-exclusion criteria

**Figure 2.**
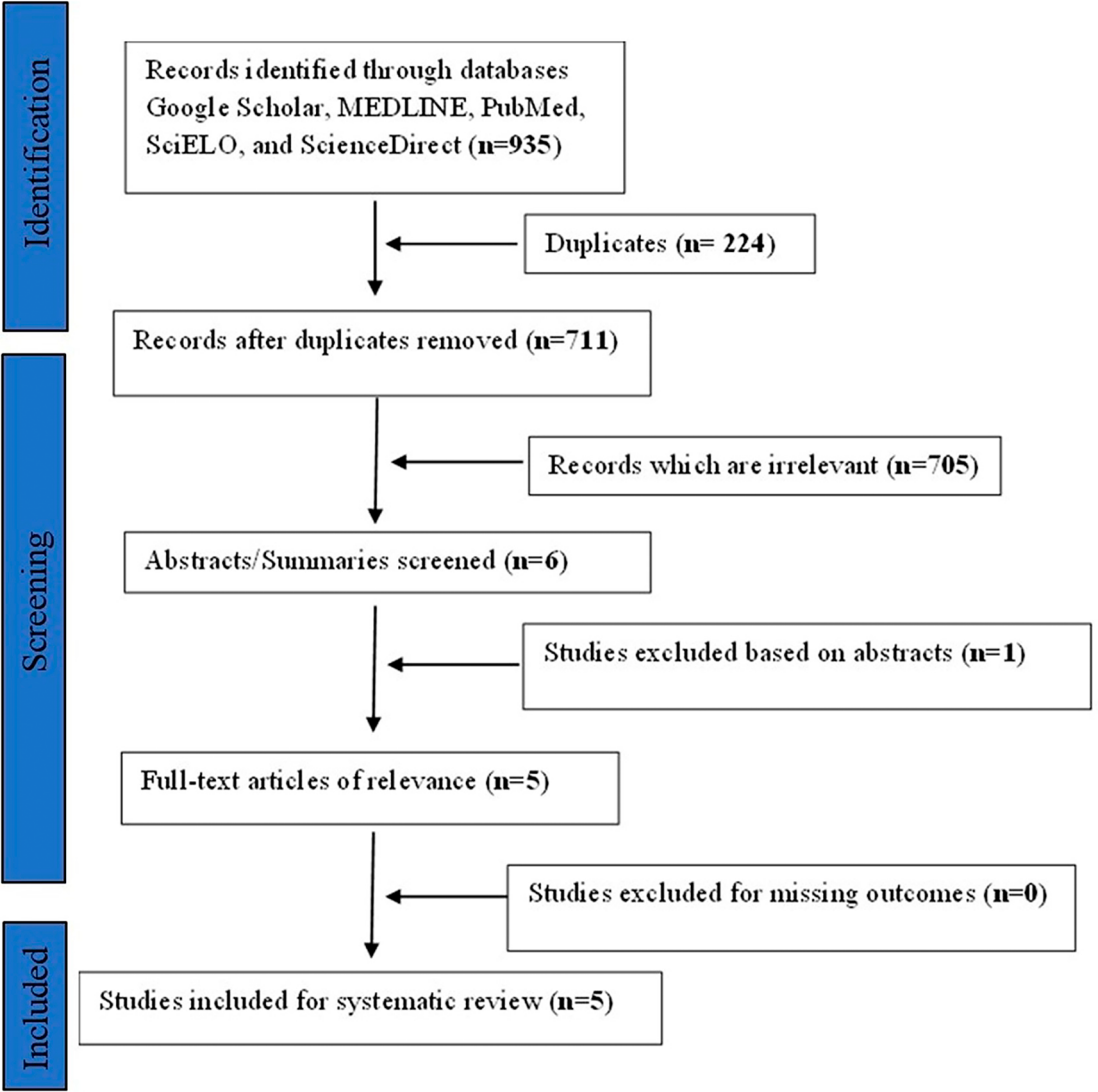
Application of selection criteria as per PRISMA guidelines.

A thorough literature search was conducted using electronic databases, such as Google Scholar, MEDLINE, PubMed, SciELO, and ScienceDirect. The keywords used for the search were “Morton’s Toe” AND “Risks” AND “Prevalence.” To arrive at a standard dataset of this biomechanically important area, we strictly confined our search criteria to the original cross-sectional studies published in English and conducted among the young adolescent and adult population by excluding all case reports, case series, letters to the editor, brief communications, and studies that did not meet the keywords of our search. Two authors (BKP and AA) independently evaluated each included article, and any disagreements were discussed and addressed by other authors (ZMHH, WANE, ANA, AAY, and MAA). The mean pooled data on the prevalence of Morton’s toe was set as the outcome of our study. The references of the included studies were thoroughly checked and duplicates were removed. The titles and abstracts of the articles were initially screened to obtain full-text articles ( Figure 2). The guidelines of Preferred Reporting Items for Systematic reviews and Meta-Analyses (PRISMA 2020) ^3^ were used to collect our data. ^4^ This study was conducted upon receiving ethical approval from the Research & Ethics Committee (REC) of the College of Medicine and Health Sciences, Arabian Gulf University (Reference no: E27-PI-02-26) and registered in the Open Science Framework (OSF) Registries (Registration: https://doi.org/10.17605/OSF.IO/BGKY2).

### Quality assessment

The quality of the studies included in the systematic review was assessed independently by two authors (BKP and AA). All studies included in this systematic review were cross-sectional studies. Therefore, the Newcastle-Ottawa Quality Assessment Scale was applied for quality assessment, as described by Wells et al. ^5^ Using this scale, the risk bias was analyzed with a tool of three domains, that is, selection criteria, comparability criteria, and outcome/exposure criteria. Every eligible study of our systematic review was scored for each domain by giving a maximum of four stars for the selection criteria, and a maximum of two stars for the comparability criteria with a maximum of three stars for the outcome/exposure criteria.

### Statistical analysis

A systematic review and meta-analysis were conducted to evaluate the prevalence of Morton’s toe. Statistical analysis was performed using R software. A random-effects meta-analysis of proportions was conducted using the meta package with the Freeman–Tukey transformation. Heterogeneity was quantitatively assessed using Cochran’s Q test, *I* ^*2*^ statistic, and tau-squared value ( *τ* ^*2*^). An I ^2^ value of >75% was considered indicative of high heterogeneity. Forest plots were generated to display the pooled prevalence with 95% confidence intervals. Statistical significance was set at p < 0.05. The study design and reporting followed the Preferred Reporting Items for Systematic Reviews and Meta-Analyses checklist to ensure methodological transparency and reproducibility.

## Results

### Studies included

**Figure 3.**
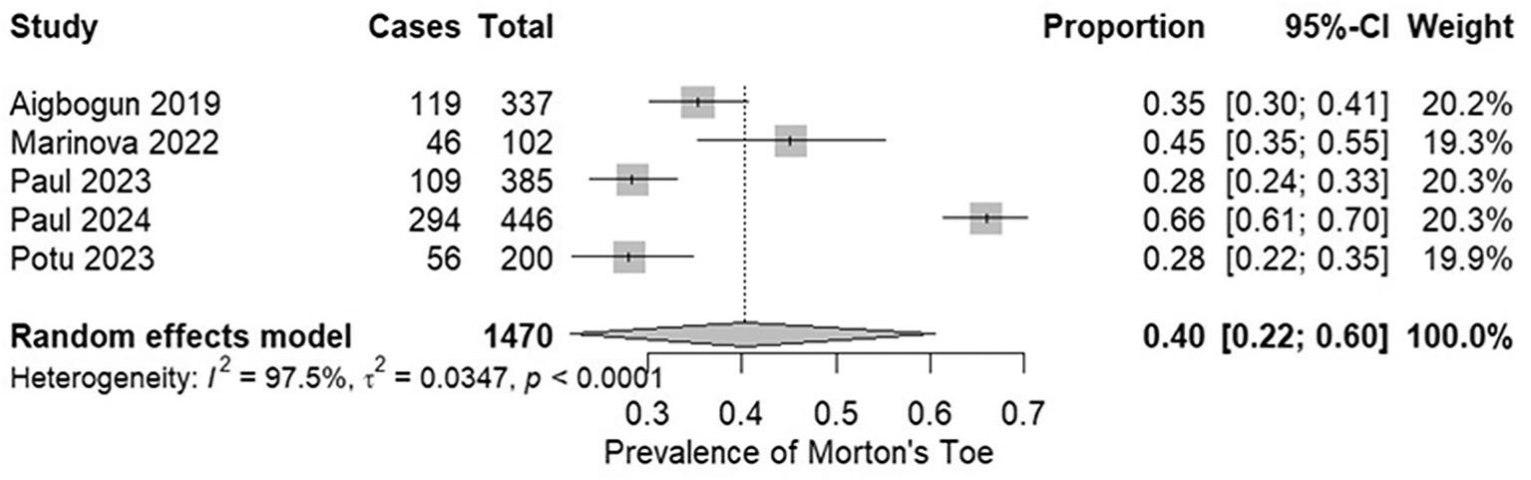
Forest plot of overall prevalence of Morton’s toe using “Random-Effects” model. CI: confidence intervals, *τ* ^*2*^ = tau-squared.

A total of five studies including 2636 feet with a sample of 1298 male, 1228 female and 110 unspecified feet met the study criteria ( Table 1 and Figure 3). Although 935 records were retrieved from the databases, only 5 studies (n = 5; 0.53% of studies) were included in our analyses because most of the studies did not report the data on keywords used in our study. The specimens included in these studies were in a range of 16–90 years age. Four studies ^6, 8– 10^ reported sex distribution in 1298 male and 1228 female lower limbs. One study ^7^ did not specify the sex of the feet. ( Tables 1, 2, 3, 4, and 5 show the characteristics of the included studies, outcomes, and quality assessment of individual studies using the appropriate Newcastle-Ottawa Scale).

**Table 1.** Studies included in our search.

Serial number	Author	Country	Year	Study design	Procedure
1	Aigbogun et al. ^6^	Nigeria	2019	Cross-sectional	Random selection, Physical observation
2	Marinova et al. ^7^	Bulgaria	2022	Cross-sectional	Random selection, Plantograms
3	Paul et al. ^8^	Nigeria	2023	Cross-sectional	Random selection, Questionnaire, Physical observation
4	Potu et al. ^9^	Bahrain	2023	Cross-sectional	Random selection, Physical observation
5	Paul et al. ^10^	Nigeria	2024	Cross-sectional	Random selection, Questionnaire, Physical observation

**Table 2.** Characteristics of the included studies.

Serial number	Author	Sample size	Sex	Age range
1	Aigbogun et al. ^6^	674	338-Male; 336-Female	NR
2	Marinova et al. ^7^	102	NR	18–60
3	Paul et al. ^8^	768	426-Male; 334-Female	18–77
4	Potu et al. ^9^	200	100-Male; 100-Female	17–75
5	Paul et al. ^10^	892	434-Male; 458-Female	16–90

NR not reported.

**Table 3.** Prevalence of Morton’s toe and associated risks.

Serial number	Author	Country	Year	Prevalence of Morton’s toe	Associated risks
1	Aigbogun et al. ^6^	Nigeria	2019	35.3%	NR
2	Marinova et al. ^7^	Bulgaria	2022	45%	Elevated medial longitudinal arch of the foot
3	Paul et al. ^8^	Nigeria	2023	28.3%	NR
4	Potu et al. ^9^	Bahrain	2023	28%	Hammertoe, metatarsalgia, bunion deformity, overpronation of the foot and plantar fasciitis
5	Paul et al. ^10^	Nigeria	2024	65.9%	NR

NR not reported.

**Table 4.** Gender-wise prevalence of Morton’s toe.

Serial number	Author	Country	Year	Prevalence of Morton’s toe	
				Male	Female
1	Aigbogun et al. ^6^	Nigeria	2019	19%	16.3%
2	Marinova et al. ^7^	Bulgaria	2022	NR	NR
3	Paul et al. ^8^	Nigeria	2023	18.7%	9.4%
4	Potu et al. ^9^	Bahrain	2023	9%	19%
5	Paul et al. ^10^	Nigeria	2024	34.7%	31.2%

NR not reported.

**Table 5.** Quality assessment of the studies based on Newcastle-Ottawa scale.

Study reference	Selection criteria ^1^	Comparability criteria ^2^	Exposure/Outcome criteria ^3^
Aigbogun et al. ^6^ (2019)	****	**	**
Marinova et al. ^7^ (2022)	***	**	***
Paul et al. ^8^ (2023)	***	**	**
Potu et al. ^9^ (2023)	****	**	***
Paul et al. ^10^ (2024)	***	**	**

^1^ Maximum of 4 stars.

^2^ Maximum of 2 stars.

^3^ Maximum of 3 stars.

** Two stars.

*** Three stars.

**** Four stars.

### Prevalence of Morton’s toe

**Figure 4.**
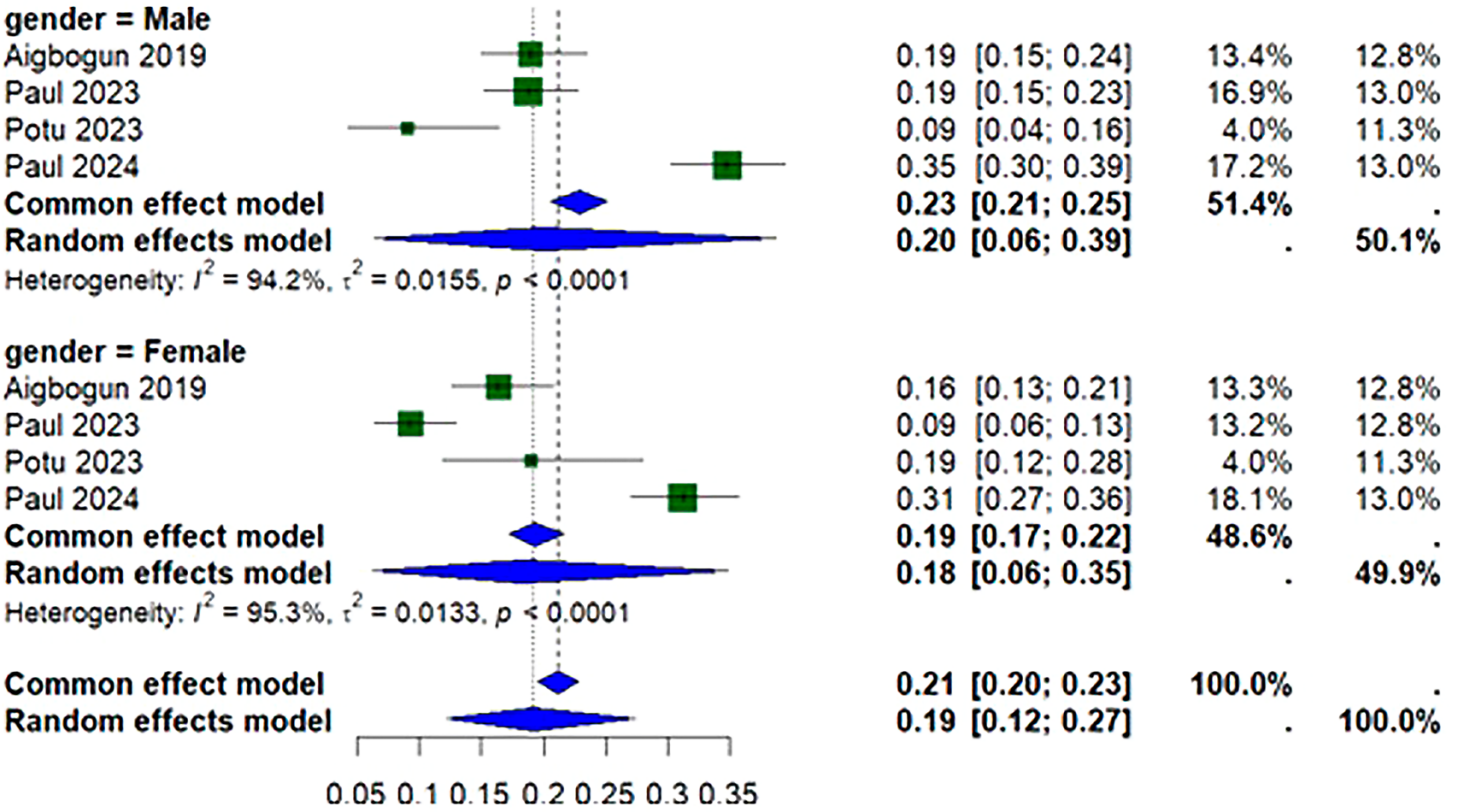
Forest plot of gender-wise prevalence of Morton’s toe using “Random-Effects” model. CI: confidence intervals, *τ* ^*2*^ = tau-squared.

Five studies reported the prevalence of Morton’s toe in different populations ( Table 3 and Figure. 3). Our pooled analysis revealed that the prevalence of Morton’s toe was 40% from different populations studied with a sample from Nigeria ^6, 8, 10^ revealed prevalence of the Morton’s toe ranging from 28.3% to 65.9% with Bulgaria sample ^7^ revealing 45% prevalence and a least being 28% from Bahraini population. ^9^ Pooled analysis on the gender-wise prevalence of Morton’s toe from five studies revealed 20.35% in males and 18.97% in females with no statistically significant differences. A sample from Nigeria ^6, 8, 10^ revealed a higher prevalence of Morton’s toe in males than the females. However, the sample from the Bahraini population ^9^ revealed a higher prevalence of Morton’s toe in females than in males ( Table 4 and Figure 4).

### Morton’s toe and the associated risks

Of the five studies, three from the Nigerian population ^6, 8, 10^ did not report any data on the associated risks of Morton’s toe ( Table 3). Two studies have reported the associated risks. ^7, 9^ Study from Bulgaria ^7^ reported that Morton’s toe is associated with elevated medial longitudinal arch of the foot, and another study from Bahrain reported multiple risks associated with Morton’s toe. ^9^ These associated multiple risks were being hammertoe, foot overpronation, bunion deformity, and plantar fasciitis.

A meta-analysis using a random-effects model demonstrated an overall pooled prevalence of approximately 40%, indicating that Morton’s toe is relatively common in the studied population. The pooled 95% confidence interval ranged from approximately 28% to 66%, reflecting variability across studies. The forest plot visually demonstrates the distribution of study-specific prevalence estimates and their confidence intervals. While several studies clustered around the pooled estimate, one study ^10^ showed a notably higher prevalence, which likely contributed to the wide pooled confidence interval and overall heterogeneity. Statistical heterogeneity among studies was extremely high, with an I ^2^ value of approximately 97.5%, indicating that most of the variability in prevalence estimates was due to real differences between studies rather than sampling error alone. The meta-analysis using a random-effects model demonstrated gender-wise pooled prevalence, as depicted in Figure 4. The significant heterogeneity may be explained by differences in geographic populations, study design, sample size, measurement techniques, and demographic characteristics of the participants.

## Discussion

The current study provides a comprehensive dataset on the prevalence of Morton’s toe in various populations, ranging from 28% to 66%. ^6– 10^ The prevalence of Morton’s toe in this study was 40% (95% CI: 22,60), with a heterogeneity of I ^2^ = 97.5%, *τ* ^*2*^ = 0.0347 (p < 0.0001). Morton’s toe, which occurs due to congenital shortening of the first metatarsal bone resulting from the premature closure of the epiphysis in the first metatarsal bone, could vary from population to population, as observed in our study. Among all the studies analyzed in our review, the sample from Nigerian populations reported that Morton’s toe could be genetically inherited, suggesting a complex inheritance pattern that does not follow simple Mendelian models, further supporting the multifactorial nature of Morton’s toe. ^6, 8, 10^ Taken together, these findings indicate that Morton’s toe likely arises from an interplay of anatomical, lifestyle, and genetic factors, emphasizing the need for a comprehensive perspective when studying or managing this condition. However, our study did not include any search for patterns of genetic inheritance.

Although Morton’s toe is considered to be a normal anatomical variant, a recent study conducted on 214 patients revealed a few cases reporting that it could lead to gait disturbances and this in turn disturbs the distribution of bodyweight causing neck pain, back pain, hip pain and knee pain, respectively. ^11^ Our previous findings clearly demonstrated that there was a statistically significant association between Morton’s toe and hammertoe (p = 0.044) and bunion deformity (χ2 = 4.069, df = 1, p < 0.001) with no statistically significant association between Morton’s toe and overpronation of foot (χ2 = 2.584, df = 1, p = 0.108); and plantar fasciitis (χ2 = 1.644, df = 1, p = 0.200). ^9^ In addition to our findings, a study from Bulgarian population showed a significant association between the high arched foot and Morton’s toe (p < 0.05). ^7^ It is important to understand how the individuals with Morton’s toe are more likely to develop associated risks such as plantar fasciitis, hammertoe and bunion deformity. Individuals with Morton’s toe overpronate their feet inward when they run or walk, which could increase the distance between the calcaneus and toes. Furthermore, this is associated with greater tension on the plantar fascia leading to plantar fasciitis and myofascial pain syndrome. ^9^ In cases of hammertoe, when inward curling of big toe occurs, the pressure and frictional force exerted on the skin and soft tissue of the first metatarsophalangeal joint. Over time, this can lead to callus formation and stress fractures. Although it is thought to be secondary to hereditary and environmental factors, a higher incidence of bunion deformity resulting from Morton’s toe is seen more in women than in men, which is believed to be due to tightly fitting women’s footwear. ^12^

Our analysis from previously published studies ^9, 10^ also suggests that the occurrence of Morton’s toe is more commonly seen in the age group of 30–45 years, particularly in females. However, our pooled analysis of the gender-wise occurrence of Morton’s toe did not reveal any significant statistical differences. Studies reported that the Morton’s neuroma resulting from anatomical variations such as Morton’s toe could be seen more in females at least five times more than males, ^13^ that to in middle-aged individuals particularly those wearing narrow and high-heeled footwear. ^14, 15^ Reported cases of Morton’s toe and Morton’s neuroma are often associated with pain exacerbated by walking with tight or heeled shoes and such pain seems to be improved by resting or wearing the appropriate footwear and Morton’s extension. ^11, 16– 18^ Morton’s extension available in the form of a flexible pad is known to raise the first metatarsal head. When the first metatarsal head is raised, the length of the great toe increases, which further increases the medial longitudinal arch. Such biomechanical changes resulting from wearing Morton’s extension pad are known to alleviate pain resulting from Morton’s toe by reducing the higher pressure on the second metatarsal. ^18^ It has been reported that increased pressure on the second metatarsal results in mid-foot arthrosis. ^19^

In conclusion, our findings provide a standard dataset on the prevalence of Morton’s toe in a systematic review and meta-analysis that has been conducted. Our findings revealed a significant association between Morton’s toe and associated risks. The results suggested that Morton’s toe prevalence varied from 28% to 66%. Despite its high prevalence, many people are unaware of Morton’s toe and its associated risks. Therefore, it is essential to raise awareness of Morton’s toe and take preventive measures for associated risks.

## Data Availability

All results and data are available in this systematic review, and no additional data sources are required. Data of PRISMA checklist is available at https://doi.org/10.6084/m9.figshare.31985214 ^4^ Figshare: PRISMA checklist for ‘Prevalence of Morton’s toe: a systematic review and meta-analysis’. https://doi.org/10.6084/m9.figshare.31985214 ^4^ Data are available under the terms of the Creative Commons Attribution 4.0 International license (CC-BY 4.0).
