# Supplementary material for: Prevalence of Morton’s toe:  a systematic review and meta-analysis
Source: F1000Res. 2026 Jun 24;15:664. Originally published 2026 May 4. [Version 3] doi: 10.12688/f1000research.180137.3 (PMC13270189; doi:10.12688/f1000research.180137.3)
Supplement: Peer review history

# Reviewer report: Reviewer response for version 2

**DOI:** 10.5256/f1000research.200988.r490597

**Published:** 2026-06-15

**Authors:** Muddanna S. Rao

**Affiliations:** 1 Department of Anatomy, College of Medicine, Kuwait University, Jabriya, Kuwait, Kuwait

The review article ‘ **Prevalence of Morton’s toe:  a systematic review and meta-analysis**’ by Zainab Mohsen Hasan Habib et al. (2026) reviewed the prevalence of Morton’s toe and its associated risks. Study is quite extensive, which included the scientific reports from all major electronic data bases and filtered the reports to five selected articles which were further reviewed. Outcome of the study revealed the interesting high prevalence of Morton’s toe in different population, its risks significance.

 Review method used and data analysis are robust, however authors need mention the statistical software(s) used for the data analysis.

 Although authors admitted that they did not include any search for patterns of genetic inheritance, it would be better to include a brief account on embryological or genetic basis of Morton’s toe in discussion

 Optionally, authors may insert the following minor edited statements for correctness and proper sequence of the statements, for better understanding.

**Abstract:**

 Background:

1. These differences in prevalence of Morton’s toe and lack of a standard dataset regarding the associated risks of Morton’s toe made us to perform a systematic review and meta-analysis on the prevalence of Morton’s toe.

Methods:

1. A systematic literature search was performed in Google Scholar, MEDLINE, PubMed, SciELO, and ScienceDirect data bases using “Morton’s Toe” AND “Risks” AND “Prevalence,” search terms adopting PRISMA guidelines.

Results:

1. Literature search yielded 935 article links, of which five studies met the inclusion criteria. These five studies comprised of 2636 feet, which were in the age range of 16–90 years.

**Main article**

 Background

 1. The foot usually consists of five toes, each having three phalanges, proximal, intermediate and distal, except the big toe, which is made up of only the proximal and distal phalanges. 

 2. Some studies even link Morton’s toe (also known as Greek toe, Royal toe) to individual’s genetics and to certain human races. 

 3.. ….while others reported low to none occurrence. 

 4.These differences in prevalence are attributed to race, ethnicity, and the different assessment methods used to quantify the frequency of occurrence. ^3–7^

 Or These differences in prevalence may be attributed to race, ethnicity, and the different assessment methods used to quantify the frequency of occurrence. ^3–7^

 5. The lack of standard dataset on the true distribution of Morton’s toe and lack of consensus between different studies makes it an excellent research topic to explore. 

 6.Moreover, studies which have investigated Morton’s toe reported many associated risks that can occur as a result of this structural variation. 

 7. As there exists variability in reported prevalence and associated risks in different studies, an extensive review of available reports was conducted and analyzed to gain a better understanding of Morton’s toe prevalence and associated risks.

**Methods:**

 1. A thorough electronic literature search was conducted on Google Scholar, MEDLINE, PubMed, SciELO, and ScienceDirect data bases.

**Results**

 1. Number of Reports and samples included for the meta-analysis

 Although 935 records were retrieved from the databases, only 5 studies (0.53% of all studies) were included in our analyses, because most of the studies did not report the data on keywords used in our study. These five studies included 2636 feet (1298 male, 1228 female and 110 unspecified) which met the study criteria (Table 1 and Figure 3). These specimens included in the studies were in the age range of 16–90 years.

 2. Five studies reported the prevalence of Morton’s toe in different populations (Table 3 and Figure. 3). Our pooled analysis revealed that the prevalence of Morton’s toe was 40% from different populations studied.  The samples from Nigeria ^3,5,7^ revealed prevalence of the Morton’s toe ranging from 28.3% to 65.9%, Bulgaria sample ^4^ revealing 45% prevalence and a least being 28% from Bahraini population. ^6^

Are the rationale for, and objectives of, the Systematic Review clearly stated?

Yes

Is the statistical analysis and its interpretation appropriate?

Yes

If this is a Living Systematic Review, is the ‘living’ method appropriate and is the search schedule clearly defined and justified? (‘Living Systematic Review’ or a variation of this term should be included in the title.)

Not applicable

Are sufficient details of the methods and analysis provided to allow replication by others?

Yes

Are the conclusions drawn adequately supported by the results presented in the review?

Yes

Reviewer Expertise:

Anatomy, Neuroscience

I confirm that I have read this submission and believe that I have an appropriate level of expertise to confirm that it is of an acceptable scientific standard.

# Response

**Published:** 2026-06-15

**Authors:** Bhagath Kumar Potu

**Affiliations:** Anatomy, Arabian Gulf University, Manama, Capital Governorate, Bahrain

Dear Reviewer

 We greatly appreciate your insightful suggestions to enhance the scientific context of our work. We incorporated all your valuable suggestions to provide a more balanced interpretation and presentation of our results. The statistical package version used in our work has been added in the manuscript with another valuable suggestion provided on the embryological background of the congenital shortening of first metatarsal bone. We appreciate the recognition of our study's methodological soundness, logical structure, and technical rigor. Your constructive feedback has helped us improve the manuscript's clarity and scientific context. We appreciate your time.

# Reviewer report: Reviewer response for version 2

**DOI:** 10.5256/f1000research.200988.r490602

**Published:** 2026-06-15

**Authors:** B.V. Murlimanju

**Affiliations:** 1 Kasturba Medical College , Manipal Academy of Higher Education, Mangalore, India

The manuscript titled 'Prevalence of Morton’s toe: a systematic review and meta-analysis' by Habib et al. is very well written and offers novel literature to the scientific community. However, I have few comments which might be addressed and this can further strengthen this manuscript.

 The following references can be cited by adding few information in the discussion section:

1. Ref 1 - (Sivaji, et al., 2013)
2. Ref 2 - (Lautzenheiser S, et al., 2013)
3. Ref 3 - (Harrison, et al., 2010)
4. Ref 4 - (Pawan., et al., 2018)
5. Ref 5 - (Pita-Fernandez, et al., 2014)
6. Ref 6 - (Ives Chatain., et al, 1986)
7. Ref 7 - (Periklis Vounotrypidis, et al., 2015) Periklis Vounotrypidis, Polyxeni Noutsou, E27. The Greek Foot: Is it a Myth or Reality? An Epidemiological Study in Greece and Connections to Past and Modern Global History, Rheumatology, Volume 54, Issue suppl_1, April 2015, Pages i182–i183, https://doi.org/10.1093/rheumatology/kev091.027

Few sentences about congenital shortening of first metatarsal bone could be included.

 The limitations of this systematic review if any can be added as the last paragraph of the discussion.

Are the rationale for, and objectives of, the Systematic Review clearly stated?

Yes

Is the statistical analysis and its interpretation appropriate?

Yes

If this is a Living Systematic Review, is the ‘living’ method appropriate and is the search schedule clearly defined and justified? (‘Living Systematic Review’ or a variation of this term should be included in the title.)

Yes

Are sufficient details of the methods and analysis provided to allow replication by others?

Yes

Are the conclusions drawn adequately supported by the results presented in the review?

Yes

Reviewer Expertise:

My area of interest in the research include Developmental anatomy of the menisci of knee joint, Neurosciences animal model research on Alzhheimer's diseases, clinical anatomy of the muscles, Morphology and Anthropology of human musculoskeletal system.

I confirm that I have read this submission and believe that I have an appropriate level of expertise to confirm that it is of an acceptable scientific standard.

## References

[1] : Morton’s Foot Syndrome: A Case Report and Overview. *Cureus* .2024; 10.7759/cureus.68731 10.7759/cureus.68731 PMC1145328439371902

[2] : Linear and Angular Measurements of the Foot of Modern Humans: A Test of Morton'S Foot Types. *The Anatomical Record* .2013;296(10) : 10.1002/ar.22764 1526-1533 10.1002/ar.22764 23963833

[3] : An exploratory study of the relationship between second toe length and androgen-linked behaviors. *Journal of Social, Evolutionary, and Cultural Psychology* .2010;4(4) : 10.1037/h0099286 241-253 10.1037/h0099286

[4] : Determination of Types of Foot in the Indian Population and Its Association with Ingrowing Toenail. *Journal of Foot and Ankle Surgery (Asia Pacific)* .2018;5(1) : 10.5005/jp-journals-10040-1082 13-15 10.5005/jp-journals-10040-1082

[5] : Podiatric Medical Abnormalities in a Random Population Sample 40 Years or Older in Spain. *Journal of the American Podiatric Medical Association* .2014;104(6) : 10.7547/8750-7315-104.6.574 574-582 10.7547/8750-7315-104.6.574 25514268

[6] : Estudio anatomo-clínico de los morfotipos digitales del antepié colombiano. *Colombia Medica* .1986;17(2) : 10.25100/cm.v17i2.6095 85-90 10.25100/cm.v17i2.6095

# Response

**Published:** 2026-06-15

**Authors:** Bhagath Kumar Potu

**Affiliations:** Anatomy, Arabian Gulf University, Manama, Capital Governorate, Bahrain

Dear Reviewer,

 We sincerely thank your compliments and constructive suggestions provided for our research. As suggested, we incorporated a brief information on the embryological background of the congenital shortening of first metatarsal bone. Additionally, we cited the suggested references in the discussion column to improve the context of our research. We appreciate your time and the thorough review process.

 Regards
